# Is Salvage Autologous Stem Cell Transplantation Still a Viable Treatment Option in Relapsed Myeloma Patients? †

**DOI:** 10.3390/medicina61050859

**Published:** 2025-05-07

**Authors:** Luka Čemažar, Matevž Škerget, Barbara Skopec

**Affiliations:** 1 Department of Hematology, University Medical Centre Ljubljana, 1000 Ljubljana, Slovenia; luka.cemazar@kclj.si (L.Č.); matevz.skerget@kclj.si (M.Š.); 2 Faculty of Medicine, University of Ljubljana, 1000 Ljubljana, Slovenia

**Keywords:** salvage autologous stem cell transplantation, relapsed multiple myeloma

## Abstract

*Background and Objectives*: The role of salvage autologous stem cell transplantation (SAT) in relapsed multiple myeloma (MM) has been increasingly questioned, particularly with the emergence of novel immunotherapies and cellular therapies. This study aimed to evaluate the efficacy of SAT in a cohort of patients with relapsed MM following their first autologous stem cell transplantation (ASCT). *Materials and Methods*: A retrospective analysis was conducted on 78 patients from our institutional registry who underwent SAT for relapsed MM between April 2008 and October 2023. The primary endpoint was the progression-free survival (PFS), with secondary endpoints including the overall survival (OS) and overall response rates (ORRs) on day +100 after SAT. *Results*: The median age of the patients was 62 years (range: 48–78), and 32% were female. Most patients (81%) received reinduction therapy before SAT. The median PFS and OS from SAT were 24 months (95% CI: 20–36) and 76 months (95% CI: 48-NR), respectively. The ORR was 85%, and 65% achieved at least a very good partial response (VGPR). No significant differences in the PFS were found between subgroups based on the maintenance following SAT (hazard ratio (HR) = 0.93, 95% CI: 0.48–1.8, *p* = 0.831), cytogenetic risk (standard vs. high-risk) (HR = 0.98, 95% CI: 0.49–1.99, *p* = 0.969), age (<60 years vs. ≥60 years) (HR = 0.96, 95% CI: 0.5–1.85, *p* = 0.912) or number of prior treatment lines (<3 vs. ≥3) (HR = 0.186, 95% CI: 0.95–3.61, *p* = 0.069). The duration of remission after the first ASCT did not influence the PFS or OS following SAT (HR = 1.63, 95% CI: 0.78–3.39, *p* = 0.193 and HR = 1.2, 95% CI: 0.46–3.09, *p* = 0.7130). *Conclusions*: Salvage autologous stem cell transplantation remains a viable treatment option for patients with relapsed MM, particularly in resource-limited countries or for patients who prefer short, fixed-duration therapy. However, as is known from previous studies, maintenance therapy is crucial for achieving longer PFS. In this study, no statistically significant factors were identified to predict the benefit, underscoring the need for further research to optimize patient selection.

## 1. Introduction

With the rapid development of novel and innovative treatment options—such as cellular therapies and immunotherapies—for patients with relapsed multiple myeloma (MM) [1], the role of salvage autologous stem cell transplantation (SAT) is increasingly being called into question. Immunotherapies including bispecific antibodies and CAR-T cell therapies are transforming the treatment landscape and challenging the role of SAT in relapsed MM. Teclistamab, a bispecific antibody targeting BCMA and CD3, demonstrated a response of at least a very good partial response (VGPR) in 58.8% of patients with a median progression-free survival (PFS) of 11.3 months in the MajesTEC-1 trial. The study included heavily pretreated patients, with a median of five previous lines of treatment and 77.6% of the patients being triple refractory [2]. Elranatamab, evaluated in the MagnetisMM-3 trial, achieved an overall response rate (ORR) in 61% of patients, with a median PFS of 17.2 months [3,4]. The median number of previous lines in this study was five, with 96.7% of the patients being triple refractory and 31.7% of the patients having extramedullary or paramedullary disease [3]. Among CAR-T cell therapies, ciltacabtagene autoleucel (cilta-cel) has been studied in the phase 1b–2 CARTITUDE-1 study in heavily pretreated patients who had received at least three lines of therapy. In the phase 3 CARTITUDE-4 study, the patients were treated with earlier lines (1–3 lines of prior therapy) and had to be lenalidomide refractory [5,6]. In the CARTITUDE-4 study, 14.4% of the patients were triple refractory and 24.0% were refractory to an anti-CD38 antibody. The ORR in patients receiving cilta-cel was 84.6%, with 73.1% achieving a complete response (CR) and measurable residual disease (MRD) negativity in 60.6% [6]. The preliminary OS findings indicated substantial improvements in earlier lines of treatment compared to the standard of care.

When considering autologous stem cell transplantation (ASCT), it is important to follow precise definitions. The European Society for Blood and Marrow Transplantation (EBMT) defined SAT as an ASCT performed in patients who experience disease progression after undergoing a previous ASCT [7]. A meta-analysis published in 2019 reported a median progression-free survival (PFS) of 13.5 months (range: 11.3–15.6) and an overall survival (OS) of 34.3 months following SAT [8]. Despite these findings, the optimal timing and indications for SAT remain subjects of ongoing discussion [9]. According to the 2021 International Myeloma Working Group (IMWG) Clinical Practice Guidelines, SAT can be offered to either transplant-naïve patients or those experiencing their first relapse/disease progression after frontline ASCT [10]. In the relapse setting, SAT may be considered if the relapse occurred after at least 36 months if maintenance therapy was used, or between 18 and 24 months in cases where no maintenance therapy was administered [11]. To date, only two randomized controlled trials have provided evidence to support the role of SAT [12,13].

The BSBMT/UKMF Myeloma X Relapse Trial demonstrated a superior time to disease progression (19 vs. 11 months) and an improved OS (67 vs. 52 months) in favor of SAT compared to weekly cyclophosphamide at 400 mg/m^2^. All patients in the study received reinduction with PAD (bortezomib/doxorubicin/dexamethasone) [13]. On the other hand, while the initial findings from the GMMG Phase III ReLApsE trial were promising [14], the results from longer follow-ups showed no significant survival benefit when comparing SAT to continuous lenalidomide/dexamethasone (RD) in relapsed and/or refractory myeloma (RRMM) [12]. Given the lack of data from prospective randomized trials, retrospective single-institution analyses have provided valuable insights. A German study led by Sellner, involving 200 patients, reported response rates of at least a partial response (PR) or better in 80% of patients by day 100 from SAT. During the follow-up period, the median PFS and OS were 15 months and 42 months, respectively, which is in accordance with previously published data [15].

Several studies (NCT05083169, NCT05572515, NCT05020236) with bispecific antibodies and CAR-T cell therapies are currently underway in earlier lines of treatment and in combination with anti-CD38 antibodies. The results will probably further challenge the role of SAT in relapsed MM. Due to the costs and limitations in availability of CAR-T therapy, there is still a place for SAT in patients in earlier lines of therapy and in those not fitting the registered indications for novel agents. Here, we report retrospective data on 78 patients receiving SAT.

## 2. Materials and Methods

The objective of this retrospective single-center study was to evaluate the efficacy of SAT in a cohort of patients with MM who experienced a relapse after an initial ASCT. We included patients from our institutional registry treated with SAT for relapsed MM between April 2008 and October 2023.

We included all patients with relapsed MM according to the IMWG criteria, older than 18 years and treated with SAT following a previous ASCT. MRD measurements were not routinely available. The only exclusion criterium was allogeneic HSCT, either prior to or post-SAT.

Demographic data on age and gender and clinical data were collected from the medical records. The disease characteristics included MM subtypes and cytogenetics (high vs. standard risk). The high-risk cytogenetic group included patients with translocations of t (4;14), t (14;16), t (14;20) deletion (del)17p, gain 1q and amplification (amp)1q. The treatment evaluation data included the number of prior lines of therapy, the type of reinduction therapy, the conditioning before and maintenance after SAT and the time to relapse from the first ASCT to SAT. The choice to proceed with either reinduction treatment or immediate SAT was the discretion of the treating physician, and was dependent on the approved drugs at the time of progression.

The treatment responses were evaluated using the IMWG uniform response criteria as a stringent complete response (sCR), complete response (CR), very good partial response (VGPR), partial response (PR) or progressive disease (PD) [16]. Only a few patients were evaluated for complete remission (CR and sCR) due to the inconvenience of performing a bone marrow biopsy and the lack of decision-making based on the results.

Immunofixation electrophoresis (IFE) was performed to detect monoclonal proteins in serum and urine. Immunotyping was utilized for the characterization of the monoclonal proteins using capillary electrophoresis. The concentrations of free kappa and lambda light chains were measured with the Serum Freelite assay (The Binding Site, Birmingham, UK).

The primary endpoint of the study was the PFS from SAT, defined as the time from the initiation of SAT to the first documented disease progression or death from any cause. The secondary endpoints were the overall response rates (ORRs), defined as the proportion of patients achieving a PR or better on day +100 post-SAT, and the OS, which was measured as the time from SAT to death from any cause.

We used Jamovi (Version 2.6; Jamovi Project, 2024) for the statistical analyses. Descriptive statistics, including the medians, standard deviations and proportions, were calculated for the continuous and categorical variables. A Kaplan–Meier survival analysis was performed to estimate the time-to-event data, and the survival curves were plotted. The differences in survival between groups were assessed using the log-rank test. Additionally, Cox proportional hazard regression models were used to evaluate the associations between the clinical variables and the survival outcomes. Hazard ratios (HRs) with 95% confidence intervals (CIs) were computed to quantify the effects of the covariates. This study complies with the Declaration of Helsinki and was approved by the local ethical committee at the University Medical Center Ljubljana (KSEV-9-030924). All patients provided written consent for data collection and analysis in our national registry and international registries for ASCT.

## 3. Results

A total of 78 patients with relapsed multiple myeloma (MM) who underwent salvage autologous stem cell transplantation (SAT) were included in the study. The median follow-ups (FUs) from diagnosis and from SAT to relapse were 87.5 months (range: 36–195) and 29 (range: 3–83) months, respectively. The median time from the first ASCT to SAT was 46 months (range: 21–163). Twenty-eight (28%) patients received maintenance therapy after the first ASCT. Most patients (81%) received reinduction therapy before SAT. The median number of prior therapy lines before SAT was two, with 26% of the patients having received three or more lines of treatment. The reinduction regimens included daratumumab-based triplets (28%), second-generation proteasome inhibitor (PI)-based triplets (27%) and other reinduction regimens incorporating bortezomib and immunomodulators. The baseline characteristics are presented in Table 1.

The conditioning regimen for SAT was melphalan, used at a full dose of 200 mg/m^2^ in 86% of cases, while a reduced dose of 140 mg/m^2^ was administered in the remaining 14% of the patients. The median age at the time of SAT was 62 years (range: 48–78), and 32% of the patients were female. Forty-eight patients (62%) received maintenance therapy after SAT (27 lenalidomide and 5 bortezomib).

**Figure 1:**
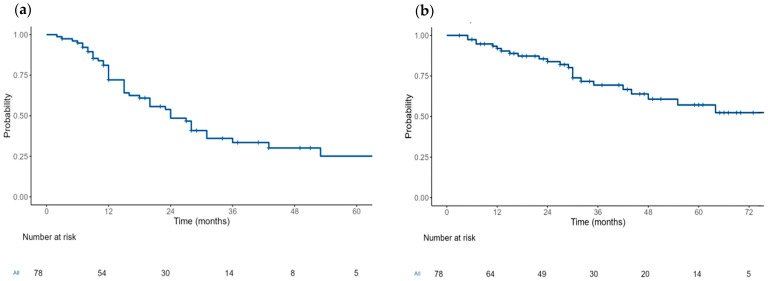
Progression-free survival (**a**) and overall survival (**b**) following salvage autologous bone marrow transplantation.

The median PFS from SAT was 24 months (95% CI: 20–36) (Figure 1). Following SAT, 66 patients (85%) achieved an ORR, and 51 patients (65%) achieved at least a VGPR. Additional information on the responses, maintenance after SAT, relapse and death is available in Table 2.

The median OS was 76 months (95% CI: 48-NR) (Figure 1). In the multivariable analysis, no significant differences in the PFS were found between subgroups based on the maintenance following SAT (hazard ratio (HR)) (HR = 0.93, 95% CI: 0.48–1.8, *p* = 0.831), cytogenetic risk (standard vs. high-risk) (HR = 0.98, 95% CI: 0.49–1.99, *p* = 0.969), age (<60 years vs. ≥60 years) (HR = 0.96, 95% CI: 0.5–1.85, *p* = 0.912) and number of prior treatment lines (<3 vs. ≥3) (HR = 0.186, 95% CI: 0.95–3.61, *p* = 0.069), respectively. Regarding the OS, no significant differences were found between subgroups based on the maintenance following SAT (hazard ratio (HR)) (HR = 1.37, 95% CI: 0.57–3.34, *p* = 0.483), cytogenetic risk (standard vs. high-risk) (HR = 0.74, 95% CI: 0.31–1.81, *p* = 0.513), age (<60 years vs. ≥60 years) (HR = 1.09, 95% CI: 0.46–2.61, *p* = 0.843) and number of prior treatment lines (<3 vs. ≥3) (HR = 1.11, 95% CI: 0.44–2.83, *p* = 0.826), respectively.

**Figure 2:**
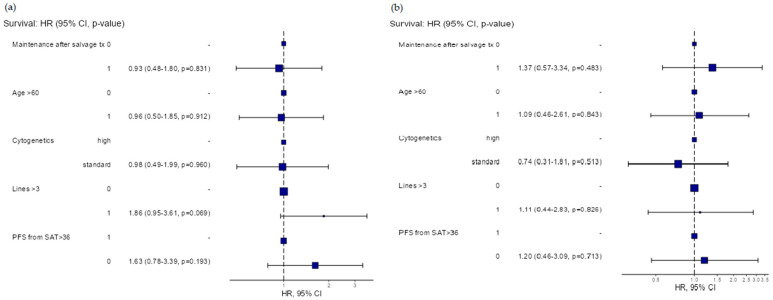
Multivariable subgroup analysis for progression-free survival (**a**) and overall survival (**b**) following salvage autologous bone marrow transplantation.

The duration of remission after the first ASCT did not influence the PFS or OS following SAT (HR = 1.63, 95% CI: 0.78–3.39, *p* = 0.193 and HR = 1.2, 95% CI: 0.46–3.09, *p* = 0.7130) (Figure 2).

## 4. Discussion

The efficacy of bispecific antibodies and CAR-T therapy has challenged the role of SAT in RR MM. We report retrospective single-center data on the efficacy of SAT in relapsed MM. As previously reported, SAT is a viable treatment option even for patients who have relapsed after multiple lines of therapy, including those refractory to daratumumab who would otherwise face a poor prognosis, with a median OS of less than 10 months [17]. It is important to note that these data were published before the widespread availability of novel therapies [18]. In our study, the ORR, PFS and OS were consistent with previously published data [19]. The long median OS of 76 months following SAT suggests that the salvage reinduction regimens, in addition to SAT, were a key factor in rescuing high-risk patients. The median PFS of 24 months in our study is difficult to compare to studies with novel agents due to the differences in the patient populations. While the results are favorable, our patient population differs significantly from those in the bispecific antibody registration studies, which included heavily pretreated triple refractory patients. The nearest comparison is to the CARTITUDE-4 study, which included patients with 1–3 prior therapies and lenalidomide refractoriness. That study showed a significant PFS improvement with cilta-cel that had not been reached at a median follow-up of 33.6 months [20]. However, cilta-cel is not approved for patients with late relapses who either did not receive lenalidomide or stopped maintenance due to side effects or MRD-guided maintenance. The access to cilta-cel remains limited, particularly in small or low-income countries, emphasizing the need for other treatment options, including SAT.

The choice of the reinduction regimen before SAT remains a key topic of discussion within both the transplant and myeloma communities [10]. A study conducted by a German group, led by Sauer, examined the outcomes of reinduction therapy prior to SAT, either as a second- or third-line therapy. The study reported a high ORR following reinduction with combination therapies involving carfilzomib and daratumumab, achieving a median PFS of 29 months [21]. Similarly, an Italian group led by Mangiacavalli focused on patients in first relapse and showed that reinduction with potent triplets such as KRd (Carfilzomib-Lenalidomide-Dexamethasone) or DRd (Daratumumab-Lenalidomide-Dexamethasone), followed by SAT, resulted in a prolonged PFS of 36.6 months [22]. The reported PFS of 24 months is in line with the data published by Sauer. Although no statistically significant differences in the multivariable analysis for the subgroups were identified, there was a shorter PFS for the patients who received more than three prior lines of treatment before SAT in the univariable analysis (HR = 1.99, *p* = 0.022). These findings suggest that SAT earlier in the disease course is of higher benefit in prolonging the PFS. The patients not receiving reinduction therapy demonstrated PFS and OS outcomes comparable to those who did. A plausible explanation is the selection of low-risk patients with biochemical progression for upfront SAT, compared to patients receiving reinduction therapy due to critical clinical progression. The long inclusion interval and the small number of patients with the various reinduction regimens used precluded an additional subgroup analysis to evaluate the potential influence of the different drugs on the SAT treatment outcomes.

More patients received maintenance therapy after SAT, as compared to after the first ASCT. This difference is attributable to the fact that at the time of the first ASCT, maintenance therapy had not been established as a standard of care and reimbursed [23]. An analysis on maintenance therapy after a second ASCT by the Center for International Blood and Marrow Transplant Research (CIBMTR) demonstrated that the 5-year median PFS rate was 27.8% for the patients on maintenance therapy, compared to 9.8% for those not receiving therapy. The 5-year median OS rate was 54% for the maintenance group versus 30.9%, further supporting the role of maintenance therapy in this setting [24]. Additional data on the role of maintenance after SAT are available in the German study, where the median PFS from SAT was 23.3 months, with the patients receiving maintenance treatment after SAT having a significantly better PFS (HR = 0.20, *p* = 0.009) [25]. Our data showed no significant benefit of maintenance therapy (HR = 0.93, 95% CI: 0.48–1.8, *p* = 0.831).

When making treatment decisions and determining the optimal treatment sequencing, we must compare the outcomes between SAT and CAR-T therapies. A European retrospective study using a large dataset found that CAR-T therapies outperformed SAT in 1-year PFS and OS. Specifically, CAR-T achieved PFS rates of 68% compared to 44% with SAT, and OS rates of 81% versus 68%, respectively. While CAR-T therapies show significant potential to improve the outcomes in heavily pretreated patients, optimal patient selection remains essential [26].

Our study has several limitations. Its retrospective nature introduces potential bias and confounding factors in selecting patients for SAT. The median time from the first transplant to SAT was 46 months, showing that SAT was primarily used for patients with a long PFS after the first ASCT. Nonetheless, we included all patients who received SAT, representing the clinical management of the patients in our practice. The small patient number limits the subgroup analysis, reducing the ability to identify a subgroup with a higher benefit of SAT. Additionally, confounding factors and family-wise type 1 errors could produce false-positive statistical results, preventing the further clarification of SAT’s impact on the subgroups. The advantage of our study is the inclusion of all patients that received SAT at our institution, reducing the possibility of reporting and referral bias.

## 5. Conclusions

Salvage ASCT remains an important treatment strategy in patients with relapsed MM with a long PFS following the first ASCT, where novel agents and CAR-T therapy are either not available or not approved.

## Figures and Tables

**Table 1:** Study population characteristics.

Characteristics	
Sex	
Male, N (%)	53 (68%)
Female, N (%)	25 (32%)
Age (range)	63 (48–74)
Cytogenetics	
High-risk	26 (36%)
Standard-risk	46 (64%)
Unknown	6
Time from first ASCT to SAT, months (range)	46 (21–163)
Reinduction therapy before SAT, N (%)	63 (81%)
Lines of therapy before SAT, N (%)	
1	13 (17%)
2	44 (56%)
3	16 (21%)
4	4 (5.1%)
5	1 (1.3%)
Maintenance after first ASCT, N (%)	
No	56 (72%)
Yes	22 (28%)

Baseline patient characteristics (sex, age, cytogenetics, maintenance, reinduction, lines of therapy). ASCT (first autologous stem cell transplantation), SAT (salvage autologous stem cell transplantation).

**Table 2:** Response to salvage autologous stem cell transplantation.

Characteristics	
Response to SAT D +100	
Progressive Disease (PD)	3 (3.8%)
Partial Response (PR)	15 (19%)
Stable Disease (SD)	9 (12%)
Very Good Partial Response (VGPR)	50 (63%)
Stringent Complete Response (sCR)	1 (1.3%)
Maintenance after SAT	
No	30 (38%)
Yes	48 (62%)
Relapse	
No	34 (44%)
Yes	44 (56%)
Death	
No	53 (70%)
Yes	23 (30%)
Lost to Follow-Up	2

SAT (salvage autologous stem cell transplantation).

## Data Availability

The data presented in this study are available on request from the corresponding author due to privacy concerns.
